# Rosai-Dorfman disease with infiltration of IgG4-bearing plasma cells presenting as laryngeal-nasal masses and cervical lymphadenopathy: A case report

**DOI:** 10.1097/MD.0000000000025165

**Published:** 2021-03-26

**Authors:** Miji Lee, Kyeong Hwa Ryu, Hye Jin Baek, Jin Il Moon, Seokho Yoon, Hyo Jung An, In Chul Nam

**Affiliations:** a Department of Radiology, Gyeongsang National University School of Medicine and Gyeongsang National University Changwon Hospital, Changwon; b Department of Radiology, Institute of Health Sciences, Gyeongsang National University School of Medicine, Jinju; c Department of Nuclear Medicine and Molecular Imaging; d Department of Pathology, Gyeongsang National University School of Medicine and Gyeongsang National University Changwon Hospital, Changwon; e Department of Radiology and Research Institute of Radiology, University of Ulsan College of Medicine, Asan Medical Center, Seoul, Republic of Korea.

**Keywords:** case report, IgG4-bearing plasma cell, larynx, nasal cavity, rosai-dorfman disease

## Abstract

**Rationale:**

Rosai-Dorfman disease (RDD) is a rare and self-limiting condition caused by the non-neoplastic proliferation of histiocytes/phagocytes in the sinusoids of lymph nodes and in extranodal tissues. Of the extranodal involvement, laryngeal involvement is extremely rare. Because of its rarity and nonspecific clinicoradiologic features, RDD is often difficult to differentiate from other benign or malignant lymphoproliferative diseases. We present a case of RDD with infiltration of IgG4-bearing plasma cells manifesting laryngeal and nasal masses with cervical lymphadenopathy.

**Patient concerns:**

A 45-year-old male patient presented with recurrent epistaxis and airway disturbance.

**Diagnoses:**

On endoscopy, there were submucosal masses in both nasal cavities and both sides of subglottic larynx. On neck CT, there were well-defined, enhancing soft tissue masses in both nasal cavities and both sides of subglottic larynx, resulting in mild airway narrowing. In addition, multiple enlarged lymph nodes showing homogeneous enhancement were noted in both parotid glands and both internal jugular chains. All lesions demonstrated marked FDG-uptake on PET/CT. Therefore, the initial radiologic differential diagnoses included lymphoma and IgG4-related disease. Biopsy was performed on the nasal and laryngeal lesions, and they revealed RDD with infiltration of IgG4-bearing plasma cells.

**Intervention:**

The patient underwent surgical resection of the masses in the nasal cavity and larynx to relieve airway narrowing.

**Outcomes:**

After surgery, airway obstruction was much improved and the patient was asymptomatic. On outpatient follow-up, he exhibited a stable condition and had no dyspnea on exercise.

**Lessons:**

Clinical awareness and suspicion are important for the accurate diagnosis and management of patients with homogeneous masses in the larynx or nasal cavity, even if there is no combined cervical lymphadenopathy.

## 1 Introduction

Rosai-Dorfman disease (RDD), also known as sinus histiocytosis with massive lymphadenopathy, is a rare non-Langerhans cell histiocytosis characterized by accumulation of activated histiocytes within affected tissues.^[1,2]^ Although pathogenesis is still unclear, the possible etiologies of this disorder are thought to include the disturbance of cell-mediated immune regulation, response to presumed infectious agents, and autoimmune mechanism.^[2,3]^ It affects mainly children and young adults, with males being slightly predominant.^[4,5]^

The major manifestation of RDD is painless bilateral cervical lymphadenopathy.^[1,6]^ Extranodal manifestation occurs in up to 43% of patients with skin, paranasal sinuses, nasal cavity, central nervous system, salivary glands, and orbit as the most commonly affected sites.^[4–9]^ However, laryngeal involvement is extremely rare, and there are less than 30 reported cases of laryngeal involvement in RDD.^[7,10]^ Because of the nonspecific imaging findings and the rarity of laryngeal involvement, the diagnosis of laryngeal RDD is difficult, which leads to inappropriate treatment of this benign but often progressive disease. Surgical resection of laryngeal RDD is recommended as it may cause life-threatening dyspnea, making early awareness of laryngeal RDD important for proper management.

In recent studies, it has been reported that a subset of patients with RDD have increased levels of IgG4-positive plasma cells on immunohistological examination. This makes differential diagnosis somewhat difficult from IgG4-related disease.^[11,12]^ Awareness of this association may lead to the correct diagnosis of RDD without being confused with IgG4-related disease.

Herein, we present a case of RDD with infiltration of IgG4-bearing plasma cells manifesting laryngeal and nasal masses with cervical lymphadenopathy. We additionally reviewed the literature to understand the clinical manifestations, image findings, pathologic features, and treatment options, which may assist in the diagnosis and proper management of RDD.

## 2 Case report

This was purely an observational case study that cannot alter the patient's management and clinical outcome. Thus, no ethical approval was required for this case report. We obtained the written informed consent from the patient for publication of this case report and accompanying images.

**Figure 1:**
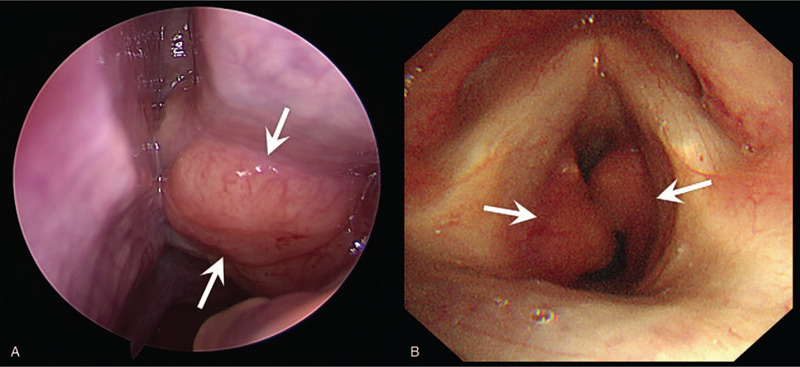
Endoscopic view of Rosai-Dorfman disease involving the nasal cavity and subglottic larynx. (A) On nasal endoscopy, there is a lobulated mass with a smooth mucous membrane in the left nasal cavity (arrows). (B) On laryngoscopy, there are nodular masses in both sides of subglottic larynx, showing smooth mucosal surface (arrows).

**Figure 2:**
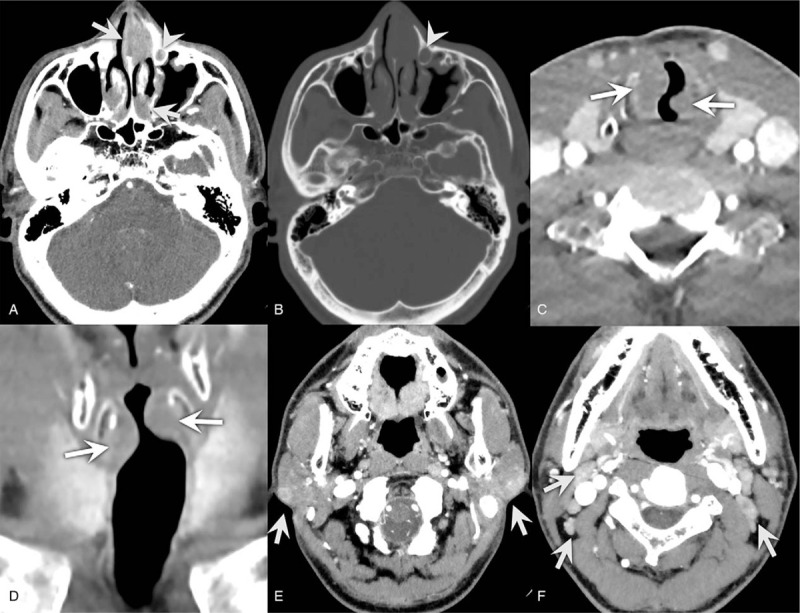
CT images of Rosai-Dorfman disease involving nasal cavities, subglottic larynx, and cervical lymph nodes. (A) Contrast enhanced CT image shows well-defined homogeneously enhancing soft tissue masses in both nasal cavities (arrows) and left nasolacrimal duct (arrowhead). (B) On bone setting image, left nasolacrimal canal is widened due to the nasolacrimal duct mass (arrowhead). (C, D) There are mildly enhancing soft tissue masses in both sides of the subglottic larynx, resulting in mild airway narrowing (arrows). (E, F) There are multiple enlarged lymph nodes showing homogeneous enhancement in both parotid glands and both level II (arrows).

**Figure 3:**
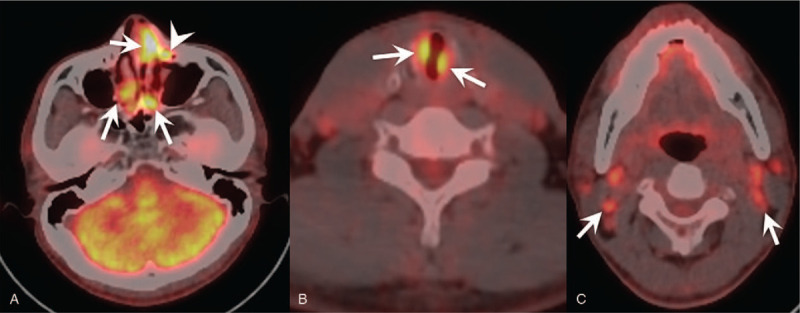
F-18 FDG PET/CT images. (A-C) There are multifocal hypermetabolic lesions in both nasal cavities (arrows on A), left nasolacrimal duct (arrowhead on A), both sides of the subglottic larynx (arrows on B), both cervical lymph nodes (arrows on C).

A 45-year-old male patient with complaints of recurrent left epistaxis for 6 months and dyspnea on exercise for 4 months. He had no medical history and there were no significant abnormalities on physical and laboratory examination. Nasal endoscopy revealed well-defined soft tissue masses in both nasal cavities (Fig. 1A). Submucosal soft tissue masses in both sides of subglottis were identified via laryngoscopy (Fig. 1B). Contrast-enhanced neck CT revealed multiple well-defined, homogeneously enhancing soft tissue masses in both nasal cavities (Fig. 2A, 2B). In addition, the enhancing soft tissue mass was opacifying left nasolacrimal canal resulting nasolacrimal canal widening (Fig. 2A, 2B). At the level of larynx, there were enhancing soft tissue masses in both sides of subglottic larynx causing mild airway narrowing (Fig. 2C, 2D). There were multiple enlarged lymph nodes showing homogeneous enhancement in both parotid glands, both infraparotid regions, level IA, IB, II, III, IV, and right level VB (Fig. 2E, 2F). On PET/CT, the soft tissue masses in both nasal cavities, left nasolacrimal duct, and subglottic larynx showed hypermetabolism (Fig. 3). Multiple enlarged lymph nodes in both parotid glands, both infraparotid region, both internal jugular chains also showed increased FDG uptake. Therefore, the initial radiologic differential diagnoses included lymphoma and IgG4-related disease.

**Figure 4:**
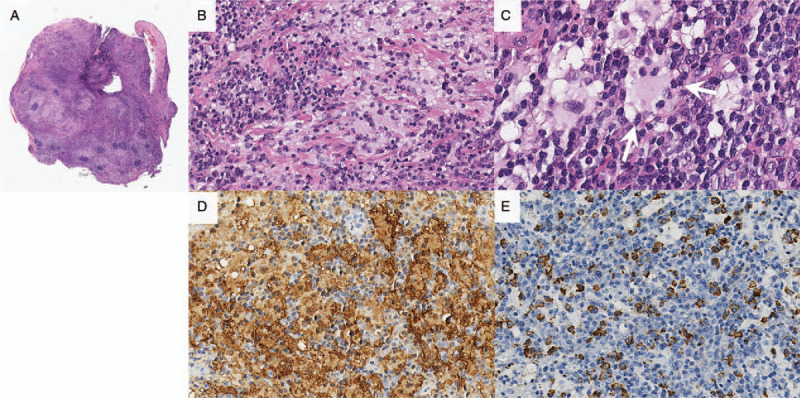
Histopathologic finding of subglottic mass. (A) The soft tissue from the subglottis is infiltrated by lymphoid cells and numerous histiocytes. There are some preserved lymphoid follicles. (x10, original magnification, Hematoxylin and Eosin) (B) On higher magnification, many histiocytes with vesicular nuclei and abundant cytoplasm are admixed with numerous plasma cells. (x200, original magnification, Hematoxylin and Eosin) (C) At the highest magnification, emperipolesis, which is the presence of intact cells within the cytoplasm of histiocytes, is shown in the center. (arrowhead). (x400, original magnification, Hematoxylin and Eosin) (D) S100P highlights the abundant cytoplasm of histiocytes, the same cells are negative for CD1a, highly suggesting Rosai-Dorfman disease. (x200, original magnification) (E) IgG4-positive plasma cells are found up to 125 per high power fields. (x200, original magnification).

Biopsy was performed for both nasal cavity masses and subglottic masses through a nasal endoscopy and bronchoscopy to achieve a confirmative diagnosis. Histopathological examination of both nasal cavity and subglottic masses showed infiltration of lymphoid cells and numerous histiocytes with preservation of lymphoid follicles (Fig. 4). Under higher magnification, emperipolesis, which is the presence of intact cells within the cytoplasm of histiocytes, was shown. Immunohistochemical staining results showed abundant cytoplasm of histiocytes expressing S-100 and CD68. The histiocytes were negative for CD1a and CD56. IgG4-positive plasma cells were also observed in the nasal cavity mass (130 per high power field) and subglottic mass (125 per high power field). The ratio of IgG4:IgG was 0.35 in the nasal cavity mass and 0.28 in the subglottic mass. Based on these findings, the lesion was histologically diagnosed as RDD with IgG4-bearing plasma cells.

After the RDD diagnosis, he had been treated with corticosteroids for several months. However, his breathing difficulty did not improve. Therefore, the patient underwent surgical excision of the masses in both nasal cavities and left subglottic larynx to relieve the airway obstruction. After surgery, airway obstruction was much improved and the patient was asymptomatic. On outpatient follow-up, he exhibited a stable condition and had no dyspnea on exercise.

## 3 Discussion

Rosai and Dorfman first described a benign histiocytic proliferative disorder in 1969.^[1,6]^ In 1972, RDD was named sinus histiocytosis with massive lymphadenopathy after performing clinicopathological analysis.^[1,6]^ Most patients with RDD are younger than 20 years (80%) with increased incidence among male patients ranging from 1.4:1 to 3:1.^[6,13,14]^ Generally, RDD involves the lymph nodes (95%), predominantly presenting as painless cervical lymphadenopathy with approximately 43% cases involving extranodal sites.^[1,6,9]^ The most common sites of extranodal involvement include the skin, paranasal sinuses, nasal cavity, central nervous system, salivary glands, and orbit.^[4–9]^ RDD patients with paranasal sinus or nasal cavity masses present symptoms of progressive nasal obstruction, recurrent epistaxis, facial pain, or hyposmia.^[7]^ RDD with laryngeal involvement, as in our case, is extremely rare; fewer than 30 cases have been reported in the English literature.^[8,10]^ The main clinical manifestations of laryngeal RDD include foreign body sensation, voice change, dyspnea, and cough.^[8,10]^ Although laryngeal RDD is a rare manifestation, awareness of laryngeal RDD can lead to early diagnosis and proper management, preventing life-threatening dyspnea. Therefore, it is recommended that RDD patients with respiratory symptoms undergo a careful laryngoscopy to exclude laryngeal involvement.

The radiologic diagnosis of RDD is often difficult because of the nonspecific imaging findings. Nodal involvement of RDD demonstrates massive bilateral cervical lymph node enlargement showing homogeneous enhancement on CT.^[7,15]^ MRI reveals homogeneous isointensity relative to the muscles on T1-wighted images and hyperintensity on T2-weighted images with homogenous enhancement.^[4,7,15]^ Differential diagnoses for this finding include lymphoma, infectious nodal disease, and metastatic lymphadenopathy.^[7,15]^ CT findings of extranodal RDD involving paranasal sinuses, nasal cavity, and larynx show homogeneously enhancing polypoid masses, mucosal thickening, or soft tissue opacification in the involved sites with or without associated bone erosion.^[7,13,15]^ On MR images, paranasal sinus lesions may show marked T2 hypointensity.^[16]^ Affected sites typically show hypermetabolism of F-18 FDG on PET/CT.^[7,15]^ Because of these nonspecific image findings, clinical suspicion is important for the accurate diagnosis of RDD in patients with homogeneous masses in the nasal cavity or larynx, even if there is no combined cervical lymphadenopathy.

The pathologic feature of nodal RDD is the sinus expansion with large histiocytes. Emperipolesis, the histopathologic characteristic of RDD, is the presence of lymphocytes, plasma cells, red blood cells or polymorphonuclear leukocytes within the cytoplasm of histiocytes.^[2,5,7,8,13]^ Extranodal involvement looks similar, but is usually associated with more fibrosis, fewer RDD histiocytes, and less emperipolesis.^[2,6,12]^ In immunohistochemical analysis, RDD histiocytes are characterized by S100 and CD68 positivity, and when combined with lymphophagocytic histocytes, give a strong indicator of RDD.^[7,13]^ The absence of CD1a immunopositivity differentiates RDD from Langerhans cell histiocytosis.^[6,17]^

In addition, as demonstrated in our case, some patients with RDD present increased levels of IgG4-positive plasma cells on immunohistological examination, making differential diagnosis with IgG4-related disease difficult.^[11,18]^ Although the number of IgG4-positive plasma cells needed to make the diagnosis, an IgG4/IgG ratio greater than 0.4 has been suggested as a more reliable and mandatory histologic criteria for the differential diagnosis of RDD and IgG4-related disease.^[12,19]^ In our case, the pathologic results demonstrated abundant emperipolesis which highly suggested RDD and the levels of IgG4-bearing plasma cells and IgG4:IgG ratio were insufficient to make a diagnosis of IgG4-related disease. Furthermore, other major histologic criteria for IgG4-related disease, such as storiform fibrosis and obliterative phlebitis, were not detected in our case. However, the significance of IgG4-bearing plasma cells in RDD remains to be further elucidated.

No uniform treatment has been established for RDD; treatment is tailored to the individual clinical circumstances.^[2]^ A variety of treatments have been applied for the management of RDD, including observation, corticosteroids, surgical resection, sirolimus, and radiotherapy. Observation is proper management for patients with uncomplicated lymphadenopathy or asymptomatic cutaneous RDD.^[2]^ However, debulking surgery may be warranted for patients with upper airway obstruction, spinal cord compression, or large lesions causing end-organ compromise, such as in our case.^[2,8]^ Therefore, clinical suspicion and early diagnosis of RDD is necessary for the selection of appropriate therapies tailored to the individual situation.

We described a rare case of RDD with extranodal involvement of the larynx and nasal cavity. Although it is extremely rare, awareness of laryngeal involvement in RDD is necessary for clinicians and radiologists as laryngeal RDD may be life threatening. Clinical awareness with meticulous pathologic review are important to provide accurate diagnosis and management in patients with homogeneous masses in the nasal cavity or larynx. We also highlight the histologic association between RDD and IgG4-bearing plasma cells even though the significance of IgG4-bearing plasma cells in RDD still remains controversial.

## Author contributions

**Conceptualization:** Kyeong Hwa Ryu, Hye Jin Baek.

**Data curation:** Miji Lee, Kyeong Hwa Ryu, Hye Jin Baek, Seokho Yoon, Hyo Jung An, In Chul Nam.

**Formal analysis:** Miji Lee, Kyeong Hwa Ryu, Hye Jin Baek, Hyo Jung An, In Chul Nam.

**Investigation:** Jin Il Moon, Seokho Yoon.

**Methodology:** Jin Il Moon, Seokho Yoon.

**Supervision:** Kyeong Hwa Ryu.

**Writing – original draft:** Miji Lee, Kyeong Hwa Ryu.

**Writing – review & editing:** Kyeong Hwa Ryu, Hye Jin Baek, Jin Il Moon, Seokho Yoon, Hyo Jung An, In Chul Nam.
